# An attempt to reverse cardiac lipotoxicity by aerobic interval training in a high-fat diet- and streptozotocin-induced type 2 diabetes rat model

**DOI:** 10.1186/s13098-019-0436-8

**Published:** 2019-06-13

**Authors:** Huan Cai, Shuchun Chen, Jingqin Liu, Yuxiu He

**Affiliations:** 1 Institute of Physical Education, Hebei Normal University, Shijiazhuang, China; 2 Department of Endocrinology, Hebei General Hospital, Shijiazhuang, China; 3 Department of Endocrinology, NO. 1 Hospital of Baoding, Baoding, China

**Keywords:** Diabetic cardiomyopathy, Cardiac lipotoxicity, Aerobic interval training

## Abstract

**Background:**

Diabetes mellitus (DM) is an important risk factor for cardiovascular disease. Aerobic interval training (AIT) has been recommended to patients as a non-pharmacological strategy to manage DM. However, little is known about whether AIT intervention at the onset of DM will reverse the process of diabetic cardiomyopathy (DCM). In this study, we sought to evaluate whether AIT can reverse the process of DCM and explore the underlying mechanisms.

**Methods:**

Fifty Wistar rats were randomly divided into a control group (CON), DCM group (DCM) and AIT intervention group (AIT). A high-fat diet and streptozotocin (STZ) were used to induce diabetes in rats in the DCM group and AIT group. Rats in the AIT group were subjected to an 8-week AIT intervention. Fasting blood glucose (FBG), lipid profiles and insulin levels were measured. Haematoxylin and eosin (HE) staining and oil red O staining were used to identify cardiac morphology and lipid accumulation, respectively. Serum BNP levels and cardiac BNP mRNA expression were measured to ensure the safety of the AIT intervention. Free fatty acid (FFA) and diacylglycerol (DAG) concentrations were analysed by enzymatic methods. AMPK, p-AMPK, FOXO1, CD36 and PPARα gene and protein expression were detected by RT-PCR and Western blotting.

**Results:**

AIT intervention significantly reduced rat serum cardiovascular disease risk factors in DCM rats (P < 0.05). The safety of AIT intervention was illustrated by reduced serum BNP levels and cardiac BNP mRNA expression (P < 0.05) after AIT intervention in DCM rats histological analysis and FFA and DAG concentrations revealed that AIT intervention reduced the accumulation of lipid droplets within cardiomyocytes and alleviated cardiac lipotoxicity (P < 0.05). CD36 and PPARα gene and protein expression were elevated in the DCM group, and these increases were reduced by AIT intervention (P < 0.01). The normalized myocardial lipotoxicity was due to increased expression of phosphorylated AMPK and reduced FOXO1 expression after AIT intervention.

**Conclusion:**

AIT intervention may alleviate cardiac lipotoxicity and reverse the process of DCM through activation of the AMPK–FOXO1 pathway.

## Introduction

Diabetic cardiomyopathy (DCM) is primarily caused by diabetes and is independent of coronary artery disease and hypertension, leading to cardiac diastolic dysfunction during the initial stage and systolic dysfunction at later stages [1]. The main pathological characteristics of DCM are suppressed glucose metabolism, elevated fatty acid metabolism and lipid accumulation within myocardial cells [2, 3]. Myocardial triacylglycerol is significantly higher in diabetes patients but is mobilized rapidly into intermediates, such as free fatty acid (FFA) and diacylglycerol (DAG) [4]. Lipid overload results in an accumulation of toxic intermediates that lead to cardiac lipotoxicity, further activating protein kinase C signalling, producing reactive oxygen species (ROS) and inducing apoptosis [5].

Because no significant clinical symptoms are observed in the early stage of DCM, it is hard to identify until patients present with heart failure. At that time, DCM is difficult to control effectively, so acting at in the early stage of DCM is crucial. Pharmacological treatment may reverse DCM in the early stage; however, pharmacological intervention will cause substantial economic pressure on the family and society [6]. Apart from pharmacological treatment, exercise intervention may also work [7]. Physical activity and exercise have been found to lower the risk of cardiovascular disease and metabolic syndrome, and regular physical activity is associated with improved longevity and reduced burden of diabetes-related complications. Therefore, exercise intervention is attracting the attention of medical and sports scientists for treating diabetes mellitus patients with heart failure [8]. Traditional exercise interventions are aimed at low-to-moderate-intensity exercise (55–70% VO2max), such as jogging and cycling [9]; however, the intensity is not high enough to stimulate adaption of the cardiopulmonary system. Aerobic interval training (AIT) consists of 1–4 min of high-intensity exercise (≥ 70% maximal aerobic capacity) with active low intensity exercise, which has been proved to improve fasting and postprandial blood glucose levels, improve VO2max, and speed up rehabilitation progress [10]. AIT is beneficial to DCM, and previous studies pointed out that it may reduce sarcoplasmic reticulum (SR) Ca2+ leak [11] and improve cardiac function [12], but few studies have directly explored the effect of AIT on cardiac steatosis.

This study aimed to investigate the effects of an 8-week AIT intervention on reducing cardiac steatosis and to further explore the underlying mechanisms involved in the reversal of DCM progression after AIT intervention.

## Methods

### DCM model induction and grouping

**Fig. 1:**
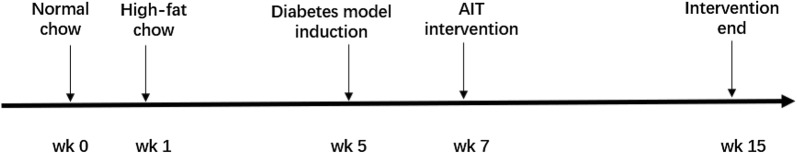
Experimental progress diagram. *DM* diabetes mellitus, *AIT* aerobic interval training

Fifty male adult Wistar rats (250–280 g, 8 weeks old) were individually housed at 20–24 °C on a 12-h light–dark cycle with free access to food and water. Ten rats were randomly selected as the non-diabetic control group (CON), and the remaining rats were used for induction of diabetes models. Diabetes was induced by 4 weeks of a high-fat diet (57.5% fat, 26.9% carbohydrate, 15.6% protein) combined with an intraperitoneal injection of 35 mg/kg STZ diluted into precooled citrate buffer solution (Sigma, St. Louis, USA) [13]. Rats in the CON group were given injections of citrate buffer alone and received a normal diet (5% fat, 53% carbohydrate, 23% protein). Fasting blood glucose levels above 11.1 mmol/L for two consecutive measurements were considered indicative of diabetes. Diabetic rats were further randomly divided into the diabetic cardiomyopathy group (DCM) and DCM with aerobic interval training group (AIT). Rats in the AIT group were subjected to an 8-week AIT intervention. All the experimental procedures were performed in strict accordance with the Guidelines on the Care and Use of Laboratory Animals as issued by the Chinese Council on Animal Research and Guidelines of Animal Care. The Ethics Committee of Hebei Normal University (Shijiazhuang, Hebei, China) approved these experiments. The experimental protocol is shown in Fig. 1.

### AIT intervention protocol

**Fig. 2:**
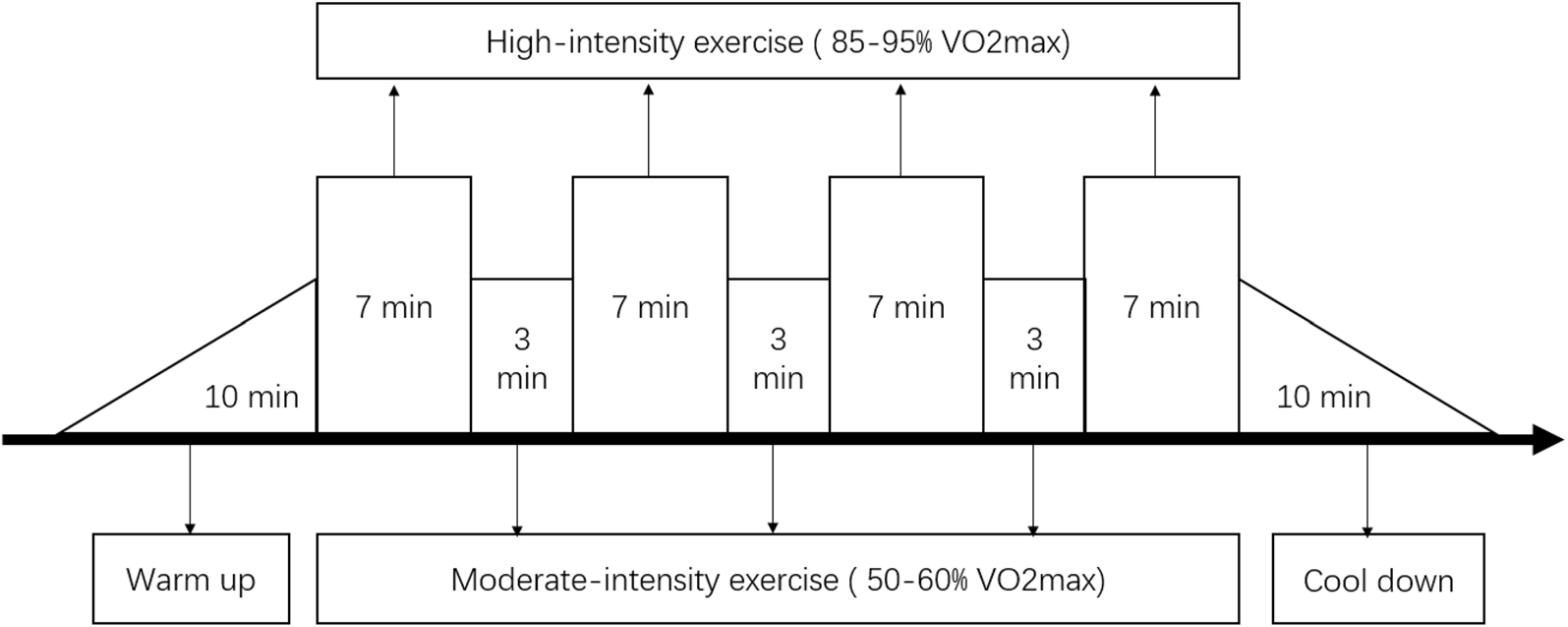
8-week AIT protocol. The frequency of AIT was 5 days per week

Rat AIT intervention was performed with a motorized rodent treadmill (ZH-PT, Zheng Hua Biologic Apparatus Facilities, Anhui, China) and consisted of a 10-min warm-up period (50–60% VO2max), four interval-training periods (7-min periods at 85–95% VO2max interspersed with 3-min intervals at 50–60% VO2max) and a 10-min cool-down period [14]. This protocol was performed once a day, 5 days a week for 8 weeks. According to Bedford’s study, we can indirectly calculate the intensity of exercise through the conversion of oxygen uptake and treadmill exercise intensity [15]. The exercise protocol is shown in Fig. 2.

### Echocardiogram examination

2D-guided M-mode images were recorded to evaluate cardiac function using a high-frequency, high-resolution digital imaging platform with linear array technology and colour Doppler mode (Vevo^®^ 2100 Imaging System, FUJIFILM Visual Sonics, Inc., Toronto, Canada). Three consecutive beats were measured, and the average of these measurements was taken for analysis. Parameters such as ejection fraction (EF), fractional shortening (FS), heart rate (HR) and cardiac output (CO) were calculated by Vevo^®^ 2100 software for calculation. The Pulse Wave Doppler mode records the peak velocity of early LV filling (E-wave) and the peak velocity of late LV filling (A-wave), which can be used to calculate the E/A ratio, as well as the isovolumic relaxation time (IVRT), E-wave deceleration time (EDT) and LV end-diastolic dimension (LVEDD).

### Blood sampling and biochemical analysis

Before sacrifice, rats underwent an oral glucose tolerance test (OGTT), and the area under the FBG × time curve was calculated. After the intervention, rats were anaesthetized with a pelltobarbitalum (35 mg/kg) intraperitoneal injection and sacrificed. Serum samples were analysed for triacylglycerol (TG), total cholesterol (TC), high-density lipoprotein cholesterol (HDL-C), low-density lipoprotein cholesterol (LDL-C) and fasting blood glucose (FBG) by enzymatic and colorimetric methods (Nanjing Jiancheng Bioengineering institute, Nanjing, China). Serum insulin and BNP levels were measured by enzyme-linked immunosorbent assay (ELISA) kits (Shanghai MLBIO Biotechnology Company, Shanghai, China). Insulin resistance was calculated according to the homeostatic model assessment of insulin resistance (HOMA-IR) with the formula HOMA-IR = glucose × insulin/22.5. HOMA-β was calculated using the following formula: HOMA-β = 20 × insulin/(glucose-3.5).

### Tissue sampling and histology

Rats were anaesthetized with pelltobarbitalum (35 mg/kg). The heart was isolated and sectioned into four slices along a plane parallel to the atrioventricular ring. The middle section of the samples was fixed in ice-cold 4% paraformaldehyde for 24–48 h, dehydrated in a concentration gradient of ethanol, embedded in paraffin and sectioned (4 μm) for haematoxylin and eosin (HE) staining. Another middle portion of the samples was used for oil red O staining to identify intramyocardial lipid deposits. The other tissue sections were rapidly dissected, frozen in liquid nitrogen, and stored in a − 80 °C freezer for further assessment.

### Measurement of cardiac FFA and DAG concentrations

Heart FFA and DAG concentrations were assayed using a non-esterified free acid assay kit and a rat diacylglycerol ELISA kit (Mlbio, China), in accordance with the manufacturer’s instructions. Five hundred milligrams of ventricular tissue zewere perfused with saline and homogenized by Grinders (IKA Tlobasic, Germany). Insoluble materials were removed by centrifugation at 4 °C for 15 min. The FFA and DAG concentrations in the supernatant were measured using an enzyme-based colorimetric assay.

### RT-PCR

Total RNA was extracted from isolated ventricular tissue using TRIzol reagent (Invitrogen, USA). First-strand cDNA synthesis was performed using a cDNA synthesis kit (Thermo, USA), and qPCR was performed using SYBR Green kits (Roche, Switzerland). The primers used in this study are listed in Table 1. The β-tubulin reaction product served as the q-PCR efficiency control. The relative transcript levels were normalized to those of β-tubulin and calculated using the 2-**CT statistical method.

**Table 1:** List of primers used for RT-PCR using SYBR Green

Gene	Length (bp)	Forward sequence (5′ → 3′)	Reverse sequence (3′ → 5′)
AMPK	153	CACTGGATGCACTCAACACAAC	TCACTACCTTCCTTCAAAGTCC
FOXO1	299	CTTCAAGGATAAGGGCGACAG	GCCATTTAGAAAACTGAGACCCA
CD36	167	AACCCAGAGGAAGTGGCAAG	GACAGTGAAGGCTCAAAGATGG
PPARα	176	TCCACAAGTGCCTGTCCGTC	CTTCAGGTAGGCTTCGTGGATT
β-tubulin	115	CGAGAAGAATACCCCGACCG	CTACCAACTGGTGGACGGAC

### Western blotting

Isolated ventricular tissues were lysed, and protein concentrations were calculated using a BCA protein assay kit. Proteins (24 µg protein per lane) were separated with 10% sodium dodecyl sulfate-polyacrylamide gel electrophoresis (SDS-PAGE) and transferred to a polyvinylidene difluoride (PVDF) membrane. The transferred protein was incubated with 5% non-fat milk buffer for 2 h at room temperature. The membranes were incubated with 1:800 anti-PPARα (Abcam, Cambridge, UK), 1:1000 anti-CD36 (Abcam, Cambridge, UK), 1:1000 AMPK (Abcam, UK), 1:1000 P-AMPK (Abcam, Cambridge, UK), 1:1000 FOXO1 (Abcam, Cambridge, UK) and 1:1000 anti-β-tubulin (Santa Cruz Biotechnology, USA) and subsequently incubated with secondary antibody (Santa Cruz Biotechnology, CA, USA). Blots were scanned and quantified using a Fusion FX multifunctional imaging system (Vilber Lourmat, France). Protein expression was calculated as the grey-scale ratio of protein/β-tubulin.

### Statistical analysis

All data are presented as the mean ± standard deviation. Statistical analysis was performed by SPSS 20.0 (IBM, New York, USA). Data were transformed using the natural logarithm to obtain a normal distribution and perform statistical analysis. One-way between-group measures ANOVA with a post hoc Bonferroni test was used to judge the differences between groups after intervention. P < 0.05 was considered to indicate a statistically significant difference.

## Results

### Characteristics of experimental rats

**Fig. 3:**
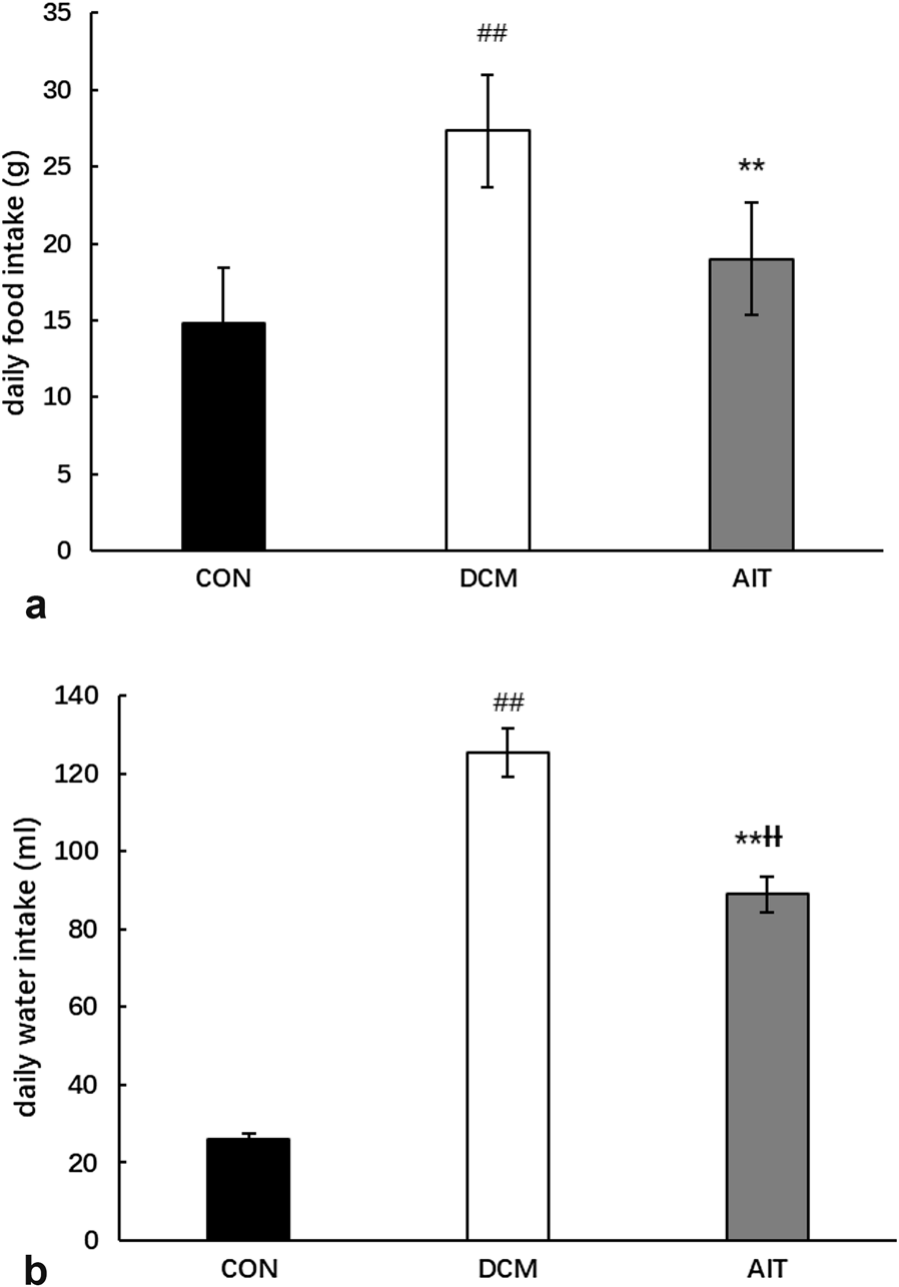
Daily food intake (**a**) and water intake (**b**) in the three groups. ^##^P < 0.01 as DCM group vs. CON group; ^ƗƗ^P < 0.01 as AIT group vs. CON group; **P < 0.01 as AIT group vs. DCM group (ANOVA)

**Fig. 4:**
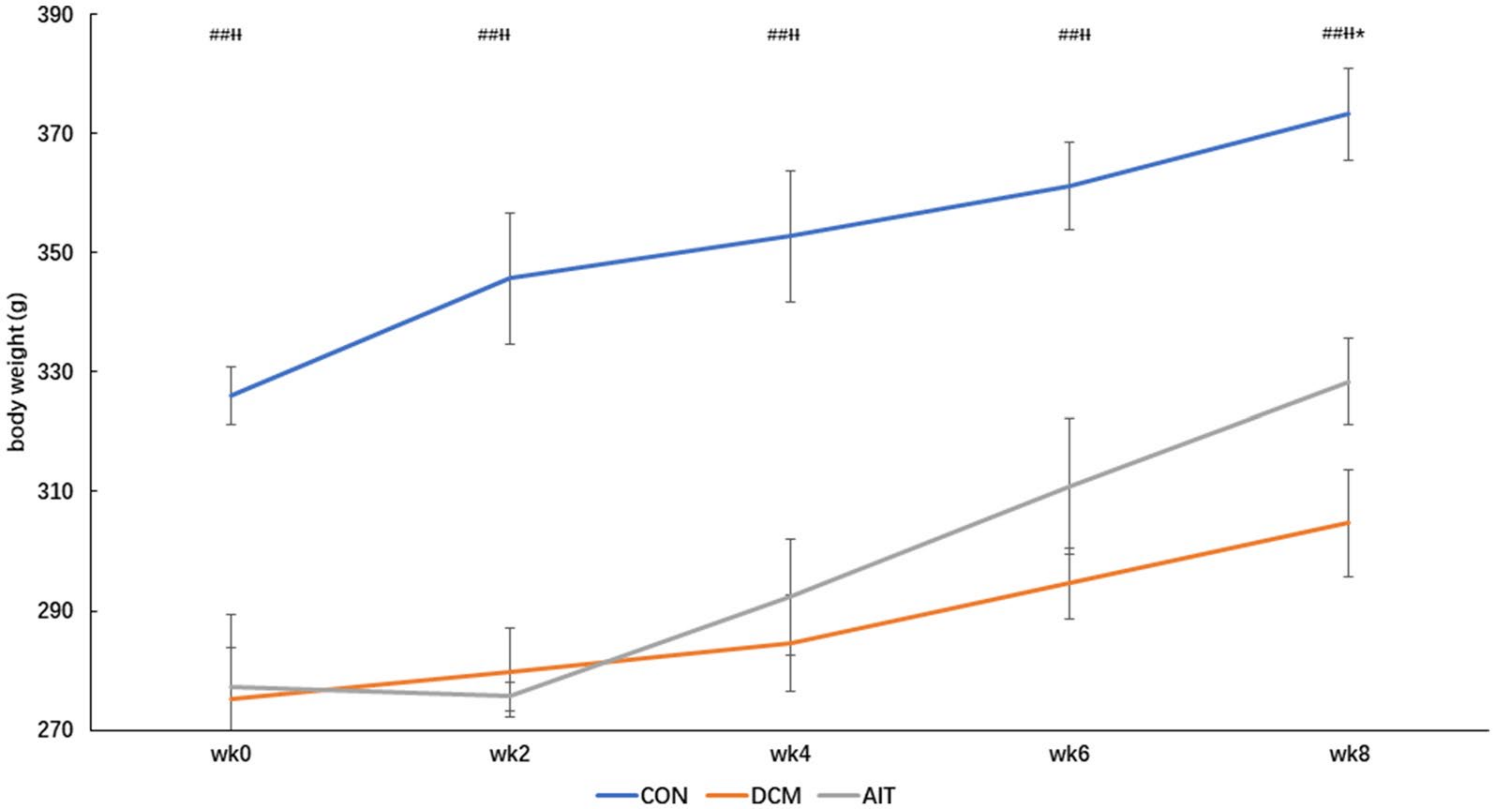
Body weight changes during the 8-week intervention. ^##^P < 0.01 as DCM group vs. CON group; ^ƗƗ^P < 0.01 as AIT group vs. CON group; *P < 0.05 as AIT group vs. DCM group (ANOVA)

After the intervention, 28 rats completed the experiment, including 9 rats in the CON group, 10 rats in the DCM group and 9 rats in the AIT group. The rats in the CON group exhibited a good mental state, and their weight increased significantly. DCM rats showed polydipsia, polyuria, and slow weight gain. The DCM modelling success rate was 48%, and the mortality rate in the DCM group was 28.6%. After 8 weeks of intervention, rats in the AIT group showed reduced food and water intake (P < 0.01) and gradually gained weight compared with rats in the DCM group. At week 8, the body weight in the AIT group was significantly higher than that in the DCM group (P < 0.05). Therefore, the characteristics of DCM rats were increased food and water intake with slowly increasing body weight, and AIT intervention reversed this phenomenon. Daily food intake and water intake are shown in Fig. 3a, b. Body weight changes among the groups are shown in Fig. 4.

### AIT improved the serum lipid profile and insulin sensitivity

Table 2 demonstrates the serum biomarker concentrations among the three groups. Compared with the CON group, DCM rats showed marked elevations in FBG, HOMA-IR, OGTT_AUC_, TG and LDL-C levels (P < 0.05); HOMA-β and HDL-C levels were decreased significantly (P < 0.05). After the 8-week AIT intervention, blood glucose parameters and the lipid profile were regulated, insulin resistance was reduced, and insulin sensitivity increased (P < 0.05). AIT intervention reversed glucose abnormalities, dyslipidaemia and insulin resistance in DCM rats.

**Table 2:** Effects of AIT intervention on serum biomarkers

	CON	DCM	AIT
N	9	10	9
FBG (mmol/L)	8.26 ± 1.48	25.22 ± 4.65^##^	13.70 ± 3.43^ƗƗ,^*
Insulin (pg/L)	2.55 ± 0.18	3.02 ± 0.29^#^	3.24 ± 0.37*
HOMA-IR	0.93 ± 0.15	3.35 ± 0.44^##^	2.01 ± 0.68^ƗƗ,^*
HOMA-β	11.88 ± 4.83	2.97 ± 1.06^##^	6.93 ± 2.16^ƗƗ,^*
OGTT_AUC_ (mmol/L)	12.29 ± 0.85	52.73 ± 4.57^##^	34.87 ± 11.71^ƗƗ,^*
TG (mmol/L)	0.76 ± 0.39	1.62 ± 0.95^##^	0.39 ± 0.12*
TC (mmol/L)	2.19 ± 0.30	2.80 ± 0.54	2.52 ± 0.34
HDL-C (mmol/L)	1.84 ± 0.07	1.13 ± 0.51^##^	1.82 ± 0.07*
LDL-C (mmol/L)	0.42 ± 0.03	1.49 ± 0.92^##^	0.66 ± 0.19*

Data are expressed as the mean ± SD

*FBG* fasting blood glucose, *HOMA-IR* insulin resistance index, *HOMA-β* β cell function index, *OGTT*_*AUC*_ oral glucose tolerance test area under the curve, *TG* triacylglycerol, *TC* total cholesterol, *HDL-c* high-density lipoprotein cholesterol, *LDL-c* low-density lipoprotein cholesterol

^#^P < 0.05 as DCM group vs. CON group, ^##^P < 0.01 as DCM group vs. CON group; ^ƗƗ^P < 0.01 as AIT group vs. CON group; * P < 0.05 as AIT group vs. DCM group (ANOVA)

### AIT improved cardiac systolic and diastolic functions

To assess the model of diabetic cardiomyopathy and whether AIT intervention may have a positive effect on cardiac function, in vivo echocardiography was performed. As shown in Table 3, DCM rats exhibited impaired diastolic and systolic functions compared with the CON group, as illustrated by the increased E/A ratio and reduced EF and FS (P < 0.05). After the 8-week AIT intervention, the E/A ratio was significantly reduced with elevated EF and FS (P < 0.05). The LVEDD and CO were similar between three groups (P > 0.05), but the HR in DCM group was significantly lower than CON group and AIT group (P < 0.05), which means DCM rats suffered impaired systolic and diastolic function, AIT intervention reversed both diastolic and systolic dysfunction in DCM rats.

**Table 3:** Effects of AIT intervention on cardiac function

	CON	DCM	AIT
N	6	6	6
LVEDD (mm)	6.39 ± 0.83	7.07 ± 0.97	6.95 ± 0.35
E/A ratio	1.09 ± 0.10	1.81 ± 0.37^##^	1.12 ± 0.04**
IVRT (ms)	29.12 ± 5.11	30.69 ± 8.62	30.92 ± 3.76
EDT (ms)	55.01 ± 3.13	73.45 ± 17.09	65.21 ± 5.60
EF (%)	80.29 ± 4.99	57.52 ± 7.22^##^	76.61 ± 4.37**
FS (%)	45.78 ± 8.68	29.87 ± 3.23^##^	44.59 ± 4.00**
HR (bpm)	346.43 ± 35.03	271.85 ± 23.84^##^	332.18 ± 60.70*
CO (ml)	58.78 ± 11.52	48.78 ± 16.16	62.57 ± 12.11

Data are expressed as the mean ± SD

*LVEDD* LV end-diastolic dimension, *E/A ratio* the ratio of E-wave and A-wave, *IVRT* isovolumic relaxation time, *EDT* E-wave decline time, *EF* ejection fraction, *FS* fractional shortening, *HR* heart rate, *CO* cardiac output

^##^P < 0.05 as DCM group vs. CON group, ** P < 0.01 as AIT group vs. DCM group, * P < 0.05 as AIT group vs. DCM group (ANOVA)

### AIT alleviated cardiac myocardial lipid contents

**Fig. 5:**
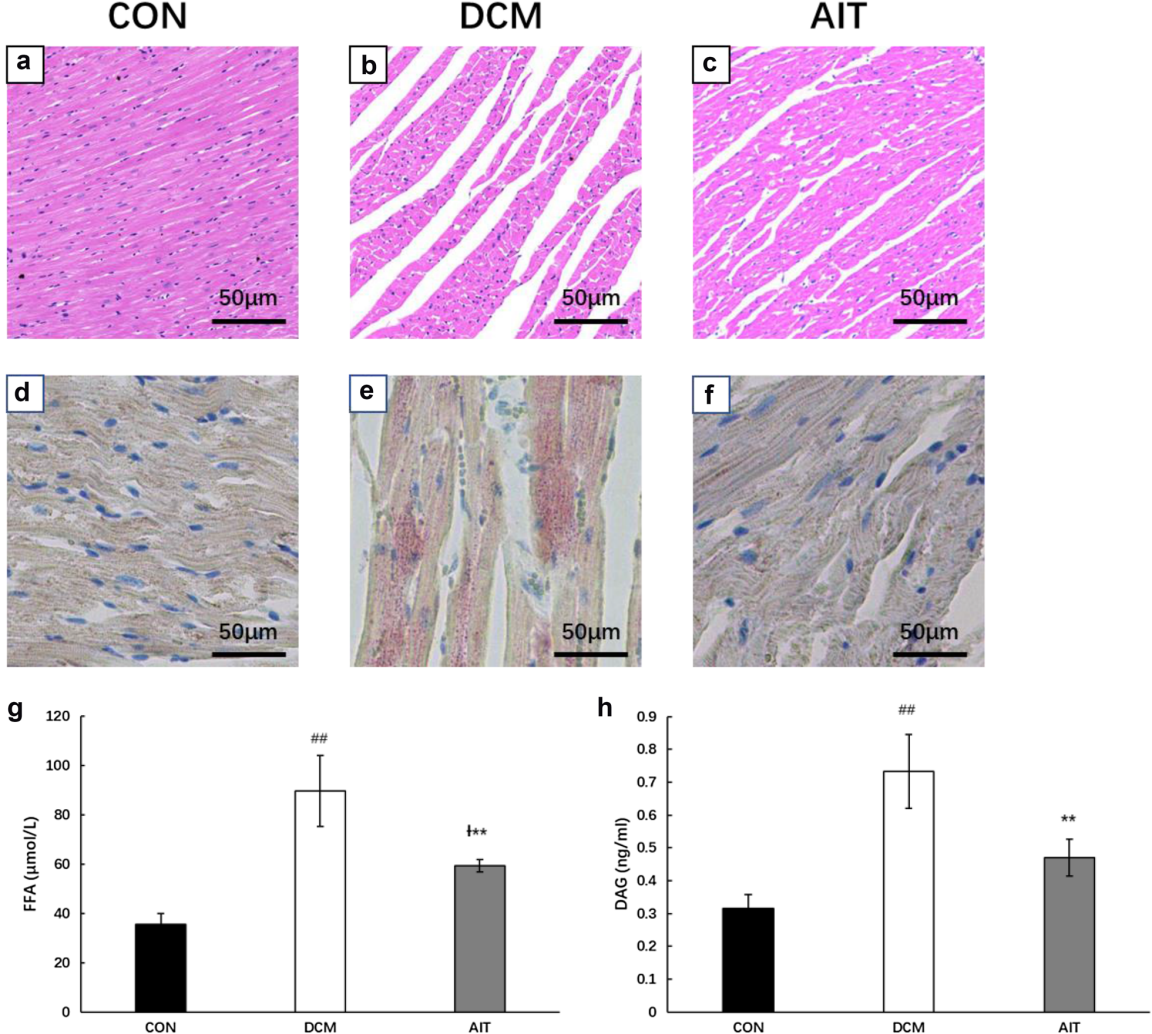
Effect of AIT intervention on lipid deposition in the hearts of diabetic rats. Tissue sections were stained with HE staining (×200 magnification) and oil red O staining (×200 magnification) to identify lipids (**a**–**f**). FFA and DAG concentrations in the hearts were normalized for tissue wet weight (**g**, **h**). ^##^P < 0.01 as DCM group vs. CON group; ^Ɨ^P < 0.05 as AIT group vs. CON group; **P < 0.01 as AIT group vs. DCM group (ANOVA)

After the 8-week AIT intervention, we evaluated myocardial lipid deposition by HE staining and oil red O staining and found disordered cardiac tissues in the DCM group and increased lipid levels within the hearts of diabetic rats; these alterations were reduced by AIT intervention (Fig. 5a–f). Consistent with the histological findings, the heart FFA and DAG contents were significantly higher in the DCM group than in the CON group (P < 0.01), and AIT intervention dramatically reduced them (P < 0.01), as shown in Fig. 5g, h. The histology results and tissue homogenate results illustrated excessive lipid accumulation within cardiomyocytes, which led to lipid steatosis. AIT intervention significantly alleviated this phenomenon.

### AIT intervention reduced PPARα-induced cardiac steatosis

**Fig. 6:**
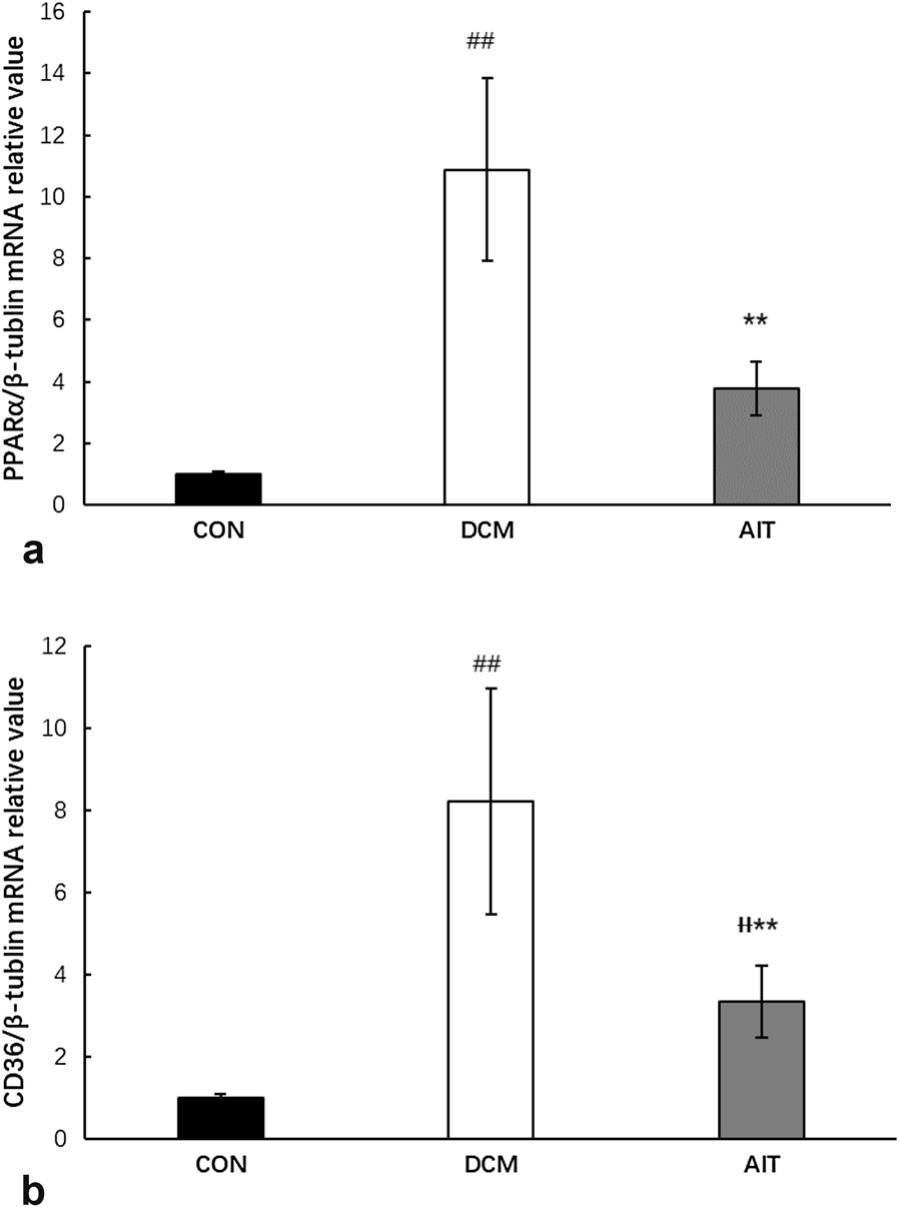
Effect of AIT intervention on relative mRNA expression of lipid metabolism-related gene PPARα (**a**) and CD36 (**b**). ^##^P < 0.01 as DCM group vs. CON group; ^ƗƗ^P < 0.01 as AIT group vs. CON group; **P < 0.01 as AIT group vs. DCM group (ANOVA)

**Fig. 7:**
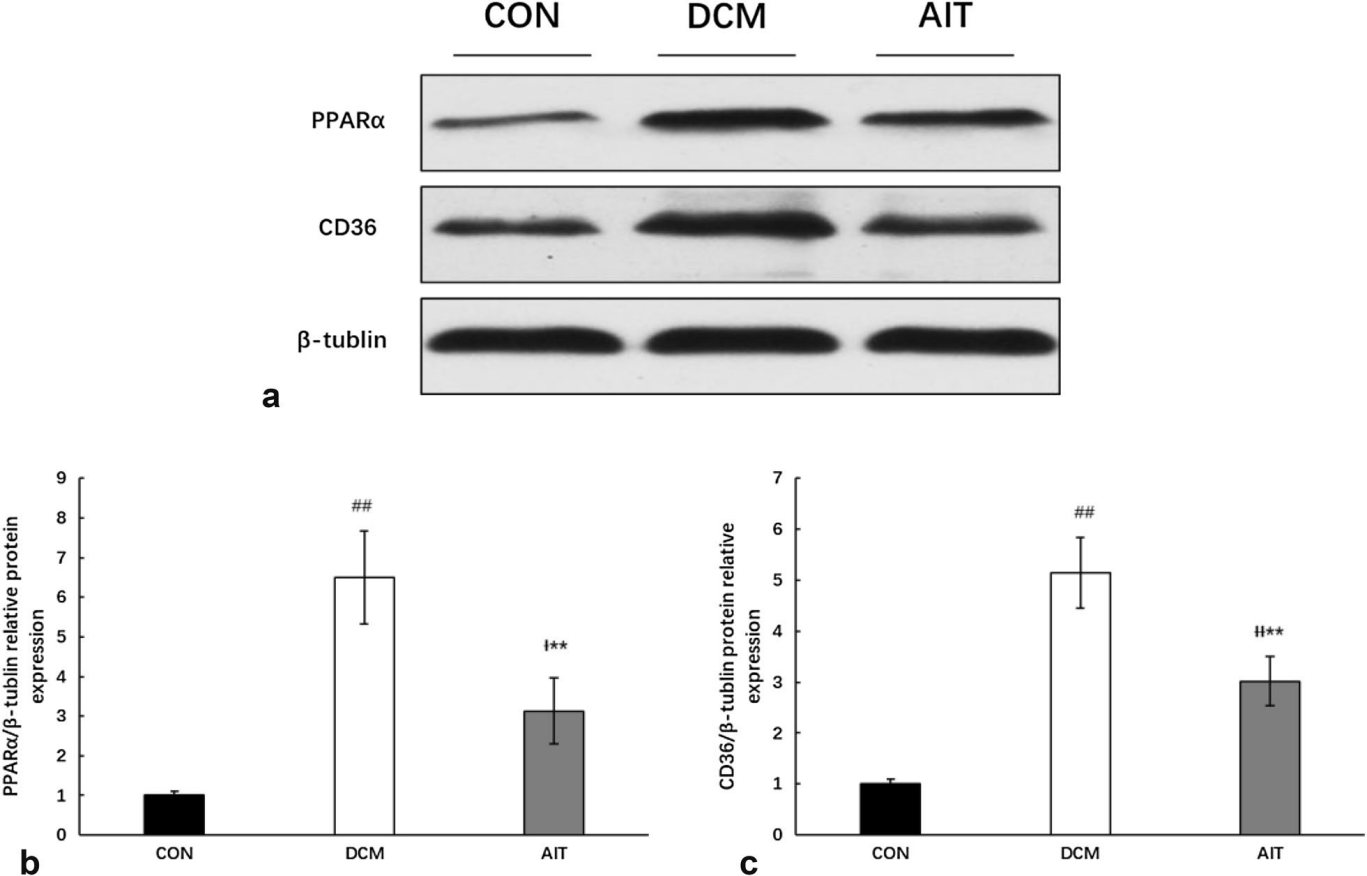
Effects of AIT intervention on relative expression of cardiac lipid metabolism-related protein. **a** The relative expression levels of PPARα and CD36 protein in the three groups were evaluated by western blotting. **b** Statistical analysis of PPARα relative expression. **c** Statistical analysis of CD36 relative expression. ^##^P < 0.01 as DCM group vs. CON group; ^Ɨ^P < 0.05 as AIT group vs. CON group; ^ƗƗ^P < 0.01 as AIT group vs. CON group; **P < 0.01 as AIT group vs. DCM group (ANOVA)

To investigate the specific mechanism of cardiac lipid deposition, cardiac lipid metabolism-related gene and protein expression levels were detected by RT-PCR (Fig. 6a, b) and Western blotting (Fig. 7a–c), respectively. As described above, myocardial FFA content was extremely high in the DCM group, activating PPARα mRNA and protein expression, compared with that in the CON group (P < 0.01). Notably, PPARα activation was inhibited by AIT intervention (P < 0.01). CD36 is a downstream factor that is regulated by PPARα; the relative expression levels of CD36 mRNA and protein were increased significantly in the DCM group compared with the CON group and dramatically decreased after the 8-week AIT intervention (P < 0.01).

### AIT intervention reverses cardiac lipotoxicity by activating AMPK–FOXO1 signalling

**Fig. 8:**
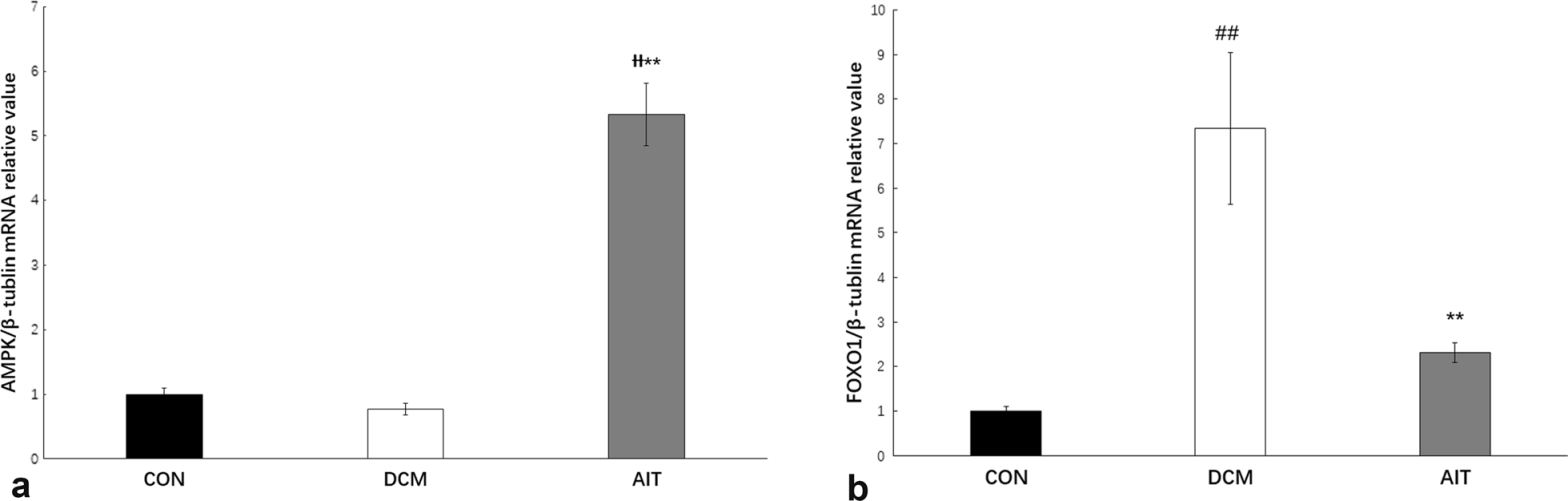
Effects of AIT intervention on relative mRNA expression of AMPK (**a**) and FOXO1 (**b**). ^##^P < 0.01 as DCM group vs. CON group; ^ƗƗ^P < 0.01 as AIT group vs. CON group; **P < 0.01 as AIT group vs. DCM group (ANOVA)

**Fig. 9:**
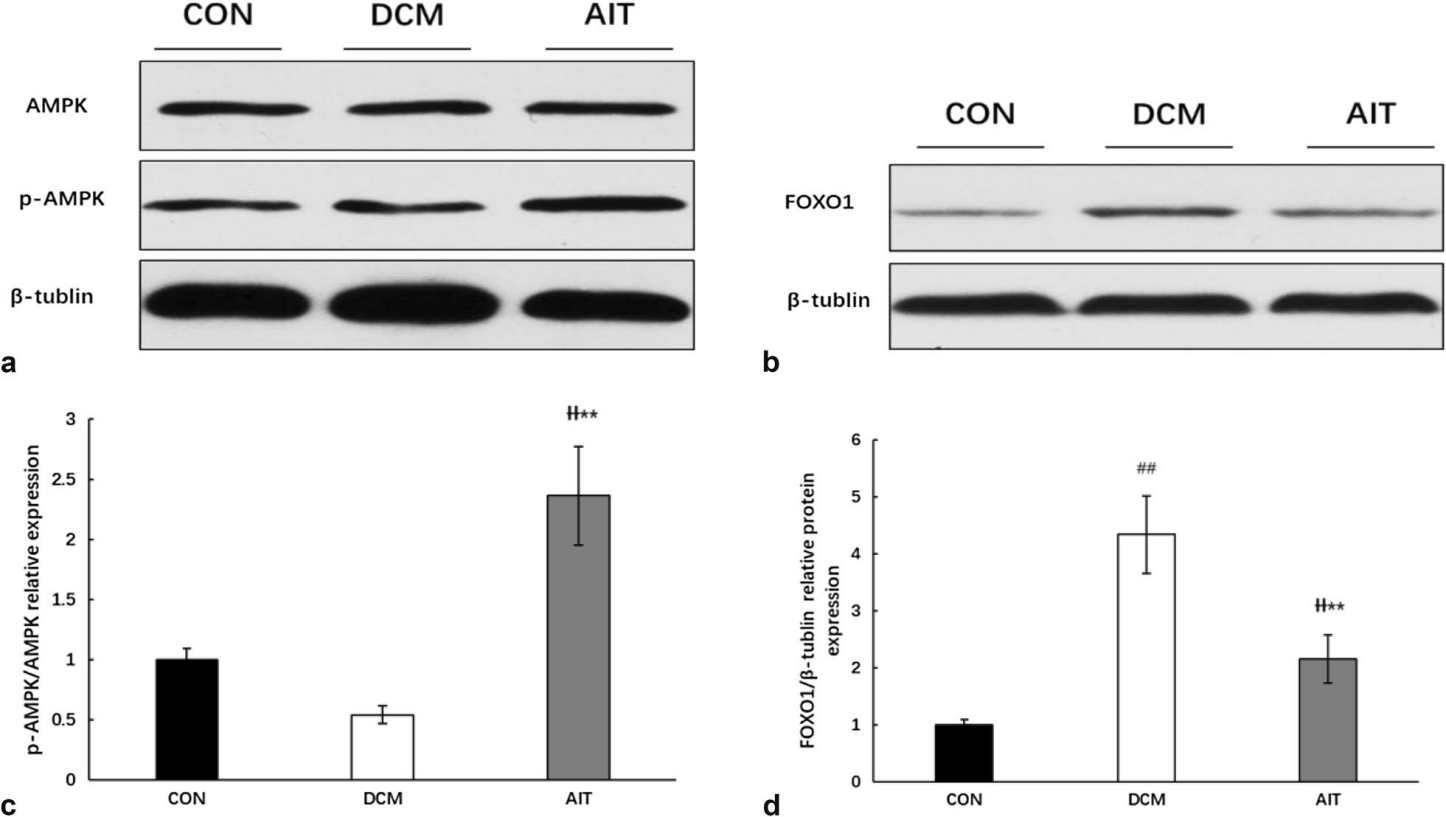
Effects of AIT intervention on relative levels of AMPK protein/phosphoprotein levels and FOXO1 protein. **a** The relative expression levels of AMPK and p-AMPK protein in the three groups were evaluated by western blotting. **b** Statistical analysis of the ratio of p-AMPK/AMPK relative expression. **c** The relative expression levels of FOXO1 protein in the three groups were evaluated by western blotting. **d** Statistical analysis of FOXO1 relative expression. ^##^P < 0.01 as DCM group vs. CON group; ^ƗƗ^P < 0.01 as AIT group vs. CON group; **P < 0.01 as AIT group vs. DCM group (ANOVA)

AMPK is a major energy-responsive receptor in mammalian cells, and it can regulate glucose uptake and FA oxidation via several mechanisms [16]. When the AMP/ATP ratio increases in cardiomyocytes, AMPK phosphorylation can control lipid metabolism [17]. As shown in Fig. 8a, the mRNA expression of AMPK was significantly increased in the hearts of the AIT group compared with those of the DCM group (P < 0.01). Western blotting analysis of AMPK and phosphorylated AMPK (Fig. 9a, b) confirmed that phosphorylated AMPK was slightly decreased in the DCM group (P > 0.05), and AIT intervention significantly increased it (P < 0.01).

FOXO1 is the downstream effector of AMPK signalling, and recent in vivo and vitro studies have indicated that FOXO1 plays a prominent role in the development of DCM [18]. As shown in Figs. 8b and 9c, d, FOXO1 mRNA and protein levels were significantly increased in the DCM group compared with the CON group (P < 0.01). After the 8-week AIT intervention, FOXO1 mRNA and protein levels were significantly decreased (P < 0.01).

### AIT intervention reduced serum and cardiac BNP mRNA expression

**Fig. 10:**
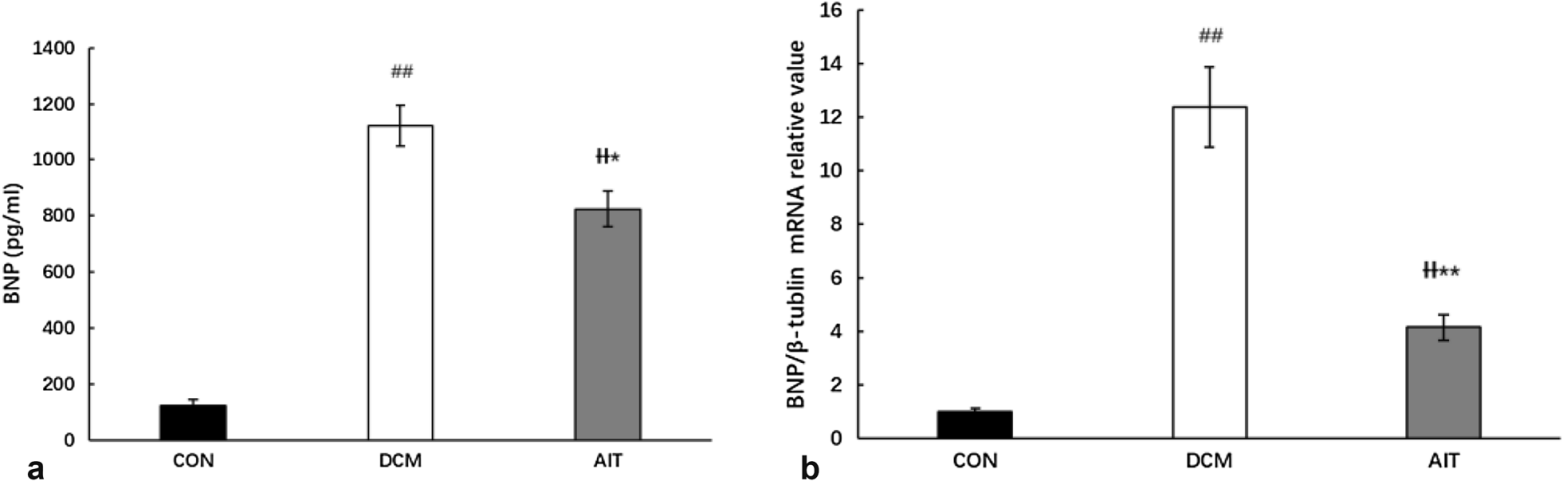
Effects of AIT intervention on serum BNP content and cardiac BNP mRNA expression. **a** serum BNP content. **b** cardiac BNP mRNA expression. ^##^P < 0.01 as DCM group vs. CON group; ^ƗƗ^P < 0.01 as AIT group vs. CON group; *P < 0.05 as AIT group vs. DCM group; **P < 0.01 as AIT group vs. DCM group (ANOVA)

To evaluate the safety of AIT intervention in diabetic hearts, we investigated the serum BNP level and cardiac BNP mRNA expression. As shown in Fig. 10a, b, the BNP levels in serum and cardiac tissue were remarkably increased in the DCM group compared with the CON group (P < 0.01). These increases in BNP levels were completely suppressed by AIT intervention (P < 0.05). AIT intervention does not lead to an increase in BNP at either the tissue or serum level; therefore, it is safe for DCM rats.

## Discussion

Numerous investigations have demonstrated that diabetes mellitus is positively correlated with cardiac dysfunction and heart failure [19]. The American Heart Association and American College of Cardiology [20] illustrated that diabetes mellitus, a grade A level heart failure risk factor, may directly damage cardiomyocytes by lipid toxicity, potentially contributing to the development of heart failure in diabetes patients even in the absence of coronary artery disease and hypertension, which is called DCM [21]. In the present study, the DCM model with increased FBG levels, insulin resistance, and impaired cardiac diastolic and systolic functions was successfully established. In addition, the HE staining results showed injured cardiac tissues, which further indicated that the DCM rats suffered structural and functional damage. This finding is consistent with previous studies and is suitable for exploring the pathologic mechanisms underlying DCM [22]. AIT intervention may significantly reverse these phenomena.

Cardiac lipotoxicity is characteristic of an accumulation of lipid droplets within cardiomyocytes [23]. Cardiomyocytes in healthy hearts have few droplets, while excessive lipids are stored in diabetic hearts [24]. In cardiomyocytes, lipids were stored in the form of TG but are rapidly mobilized into FFA. TG is non-toxic, and its intermediates, DAG and ceramides, of the non-oxidizing pathway are toxic [25]. This result is consistent with our study, in which histology and cardiac tissue results indicated that DCM hearts accumulate lipid drops inside cardiomyocytes, accompanied by high levels of FFA and DAG. AIT intervention attenuated the levels of cardiac FFA and DAG, therefore reducing cardiac lipid deposition and steatosis.

Cardiac lipotoxicity could be due to increased myocardial FFA uptake. FFA can be taken up by cardiomyocytes in two major ways: 20% of FFA is absorbed by passive diffusion, and 80% of FFA by protein-mediated transport [26]. From the serum lipid profile measurements, we can conclude that DCM rats have higher TG and LDL-c levels, which increase the source of cardiac lipids. Long-chain fatty acids (LCFAs) can be taken up through the LCFA translocase protein CD36 [27]. In the present study, we found that cardiac CD36 expression was elevated in the DCM group. CD36 takes up LCFAs into cardiomyocytes, and inner-membrane LCFA can activate PPARα, which may result in transcriptional upregulation of the enzymes involved in FA transport (FAT/CD36) and FA oxidation (CPT-1), which in turn aggravate cardiac lipid disturbance [28]. In the present study, increased FFA levels and PPARα expression were observed in DCM rats in parallel with the upregulation of CD36. Exercise has beneficial effects on regulating cardiac lipid disturbance. Notably, Chen’s study, which subjected diabetic rats to an 8-week swimming intervention, found that heart PPARα and CD36 mRNA levels were decreased and lipid toxicity was relieved by the intervention [29]. This result is consistent with our study, which showed that an 8-week AIT intervention significantly reduced PPARα and CD36 expression which restricted the source of cardiac steatosis.

AMPK, a heterotrimeric enzyme activated by an increase in the AMP/ATP ratio within cardiomyocytes, is a metabolic regulator that is involved in glucose and lipid metabolism [30]. Recently, AMPK has become a target for treating diabetes, and exercise may significantly activate AMPK signalling. Furthermore, aerobic exercise may reverse insulin resistance by activating AMPK signalling in skeletal muscle [31]. AMPK may directly phosphorylate and activate the nuclear receptor farnesoid X receptor (FXR) in the liver to promote metabolic homeostasis [32]. In addition, AMPK may modulate cardiac FFA metabolism by regulating CPT-1 through phosphorylation of acetyl CoA carboxylase (ACC) and release of the malonyl-CoA-mediated inhibition of CPT-1 [33]. Can AMPK modulate the uptake of FFA? Hay et al. proposed that the FOXO-AMPK-mTOR signalling pathway plays a key role in signal transduction in mammalian cells [34]. Metformin can inhibit the growth of oestrogen-dependent endometrial cancer cells by activating the AMPK–FOXO1 signalling pathway [35]. Moreover, AMPK–FOXO1 signalling may also regulate proteolysis in rat cardiomyocytes [36]. The FOXO transcription factor family includes FOXO1, FOXO3, FOXO4 and FOXO6. These transcription factors participate in stress response, protein hydrolysis, apoptosis, etc. [37]. Forkhead box protein O1 (FOXO1) has been reported to participate in abnormal myocardial metabolism in vivo and in vitro [18]. Interestingly, FOXO1 is overexpressed in the diabetic heart, and the FOXO1-iNOS-CD36 axis was reported to be involved in enhancing fatty acid flux into cardiomyocytes [38]. FOXO1-knockout mice also showed inhibition of CD36 gene expression [39]. Furthermore, fenofibrate reduces lipid accumulation in myotubes by modulating the PPARα/AMPK/FOXO1/ATGL pathway [40]. This finding is consistent with our results in the present study showing that improved AMPK phosphorylation after AIT intervention significantly reduced cardiac FOXO1 expression, in parallel with decreased CD36 expression and reduced PPARα expression, eventually reducing cardiac steatosis.

Low cardiorespiratory fitness is a risk factor for DCM patients, and exercise training is the most effective and costless way to increase cardiorespiratory fitness [41]. Traditional exercise focused on low-to-moderate-intensity exercise, while more vigorous exercise may be associated with cardiovascular risk [9]. Vered’s study pointed out that acute exercise may induce ventricular dysfunction in young men with asymptomatic diabetic cardiomyopathy [42]. Thus, the safety of exercise for DCM patients is crucial. BNP is a hormone that is secreted as a reaction to ventricular distention and stretching [43]. Increased serum BNP concentrations indicate cardiac hypertrophy and diastolic dysfunction. In our study, DCM rats showed increased serum and cardiac BNP levels. However, the effect of exercise on BNP levels is controversial. Our results illustrated that AIT training also reduced BNP levels, which contrasts with Hsfstad’s research, which pointed out that both high-intensity and moderate-intensity training normalized left ventricular (LV) diastolic and systolic function but did not change BNP levels in obese C57BL/6J mice [44].

## Conclusions

In conclusion, this study is the first to demonstrate that AIT intervention may reverse diabetic cardiomyopathy at the onset against myocardial steatosis, at least in part, by activation of AMPK–FOXO1 signalling.

## Data Availability

The datasets used and/or analysed during the current study are included in this published article.

## Abbreviations

ACC: CoA carboxylase
AIT: aerobic interval training
AMPK: adenosine 5′-monophosphate -activated protein kinase
BNP: B-type natriuretic peptide
CD36: cluster of differentiation 36
CPT-1: carnitine palmitoyl transferase I
DAG: diacylglycerol
DCM: diabetic cardiomyopathy
FBG: fasting blood glucose
FFA: free fatty acid
FOXO1: Forkhead box protein O1
FXR: farnesoid X receptor
HDL-c: high-density lipoprotein cholesterol
HOMA-IR: insulin resistance index
HOMA-β: β-cell function index
HR: heart rate
LCFA: long-chain fatty acid
LDL-c: low-density lipoprotein cholesterol
LVEDD: LV end-diastolic dimension
OGTT: oral glucose tolerance test
p-AMPK: phosphorylation of AMPK
PPARα: peroxisome proliferator-activated receptor alpha
STZ: streptozotocin
TC: total cholesterol
TG: triacylglycerol
T2DM: type 2 diabetes mellitus
